# Supplementary material for: Room-temperature oxygen vacancy migration induced reversible phase transformation during the anelastic deformation in CuO
Source: Nat Commun. 2021 Jun 23;12:3863. doi: 10.1038/s41467-021-24155-z (PMC8222270; doi:10.1038/s41467-021-24155-z)
Supplement: Supplementary file 1 — Supplementary Information [file 41467_2021_24155_MOESM1_ESM.pdf]

Supplementary Information for

Room-temperature oxygen vacancy migration induced reversible phase transformation during the anelastic deformation in CuO

Lei Li[1‡], Guoxujia Chen[1‡], He Zheng[1,2,3*], Weiwei Meng[1], Shuangfeng Jia[1], Ligong Zhao[1], Peili Zhao[1], Ying Zhang[1], Shuangshuang Huang[1], Tianlong Huang[1] & Jianbo Wang[1*]

[1]School of Physics and Technology, Center for Electron Microscopy, MOE Key Laboratory of Artificial Micro- and Nano-structures, and Institute for Advanced Studies, Wuhan University, Wuhan 430072, China

[2]Suzhou Institute of Wuhan University, Suzhou, Jiangsu 215123, China

[3]Wuhan University Shenzhen Research Institute, Shenzhen, Guangdong 518057, China

Corresponding to: wang@whu.edu.cn (J. B. W.); zhenghe@whu.edu.cn (H. Z.)

This file includes:

Supplementary Figures 1-22

Supplementary Table 1

Supplementary References

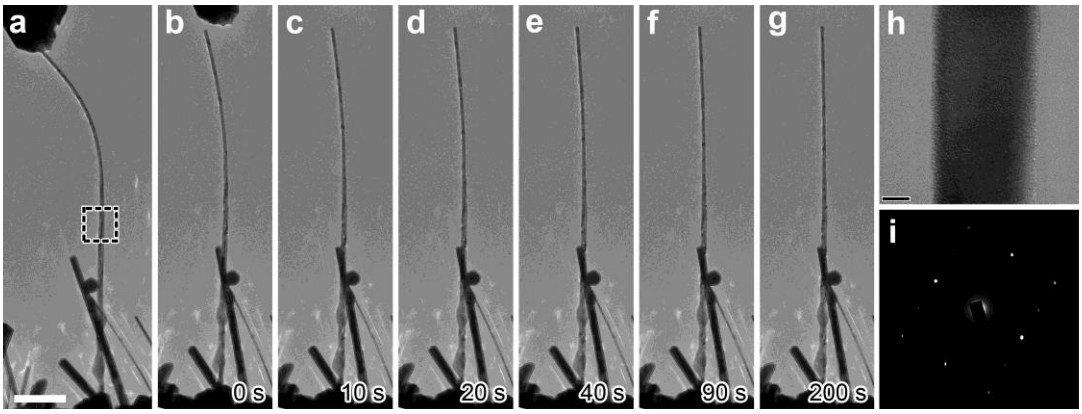

Supplementary Figure 1 | The anelastic behavior in a single crystalline CuO NW. **a**-**g**, Low-magnified TEM images showing the shape recovery of a CuO NW. **h**, Magnified view of enclosed area in **a**. **i**, Selected area electron diffraction (SAED) pattern obtained from **h** indicating the single crystalline structure of the NW. Scale bar, 500 nm (**a**-**g**), 10 nm (**h**).

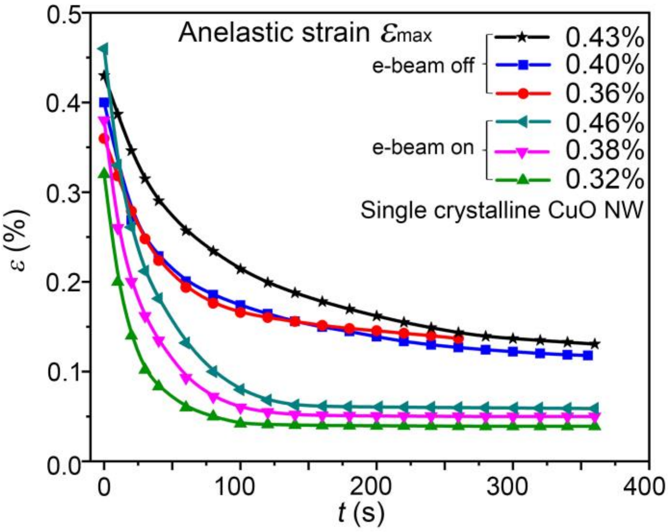

Supplementary Figure 2 | E-beam irradiation effect on the anelasticity in a single crystalline CuO NW. Anelastic strain recovery as a function of time in three bending tests with and without e-beam irradiation on the same single crystalline CuO NW, respectively. Without e-beam irradiation, the time-interval for the image recording is 20 seconds.

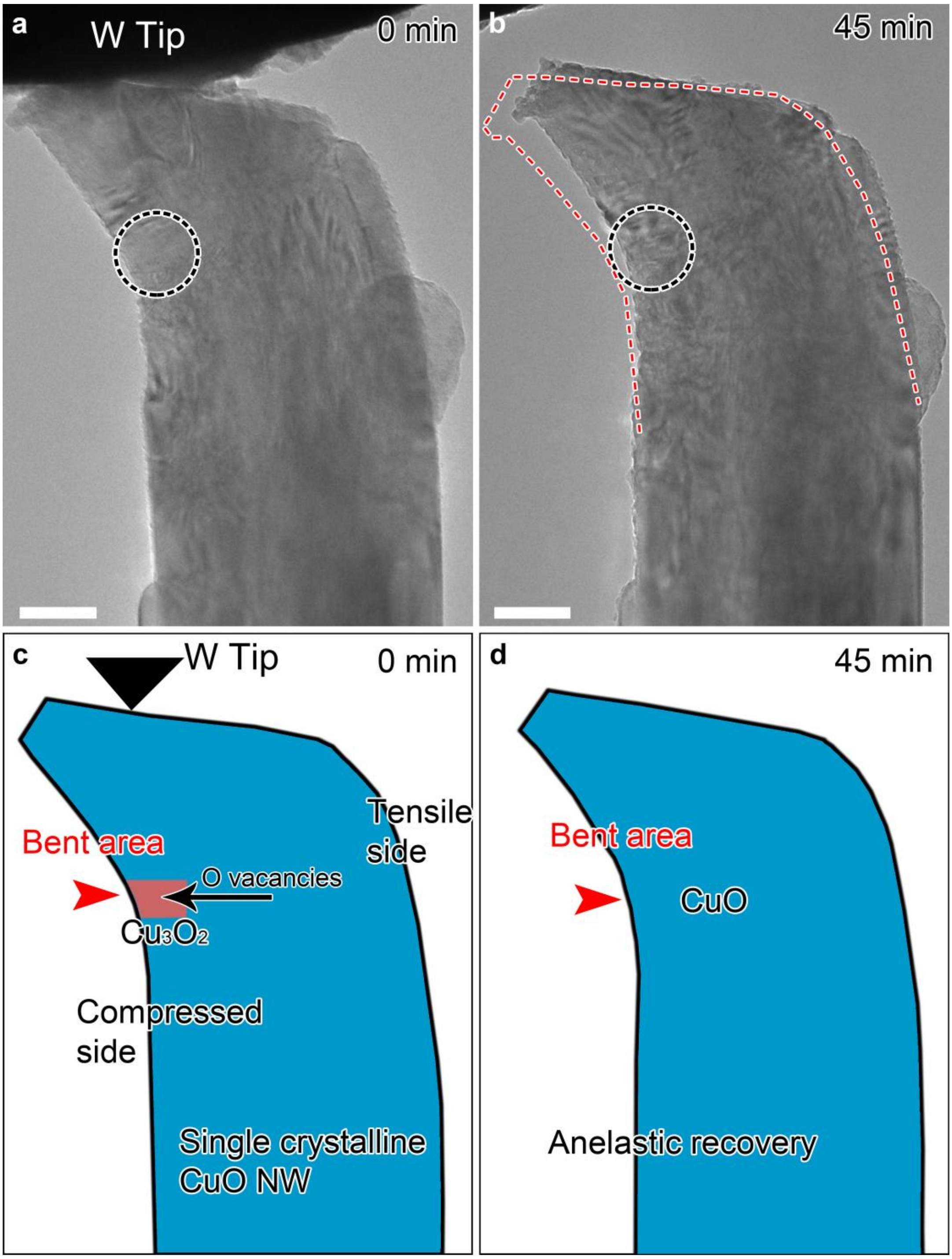

Supplementary Figure 3 | The anelastic strain recovery in a single crystalline CuO NW. a,**b**, Low-magnified TEM images showing the shape recovery of a CuO NW after the bending stress released. **c**, Nucleation of CuO_x phase at the NW compressive side, which gradually transforms back to CuO (**d**). Scale bar, 50 nm (**a**,**b**).

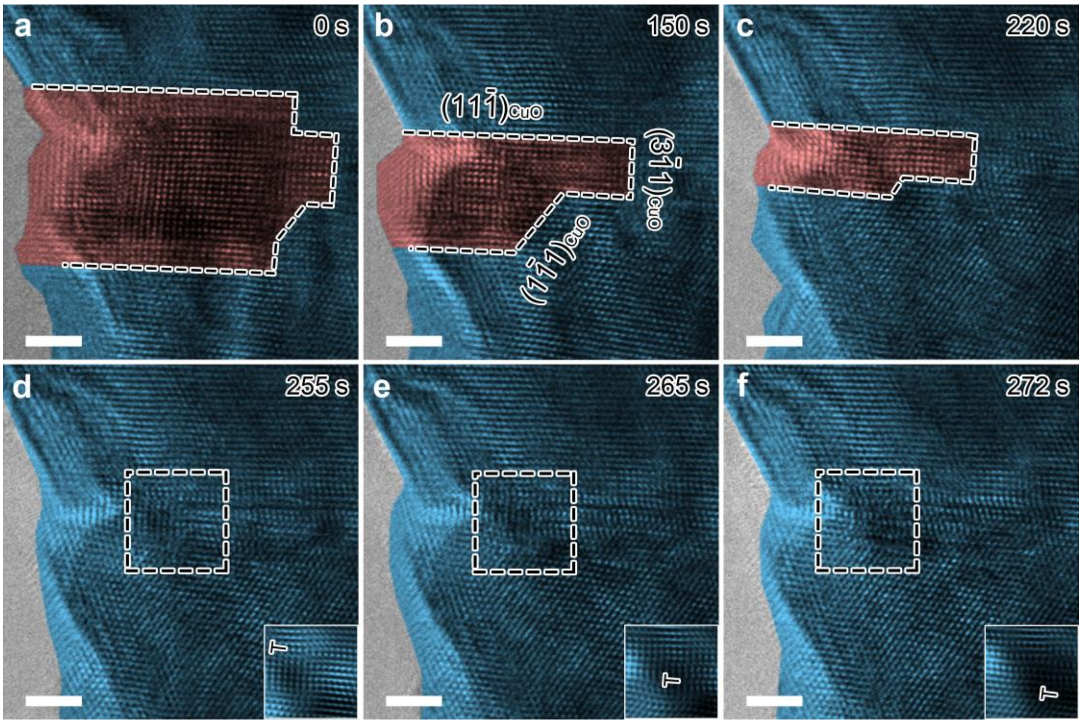

Supplementary Figure 4 | The atomistic structural evolution during the CuO_x-CuO phase transition. a-f, HRTEM images illustrating the phase boundary evolution as pointed out by the dotted lines. Fourier filtered images of the boxed areas in **d-f** shows the evolution of dislocation during the phase transition. Scale bar, 2 nm.

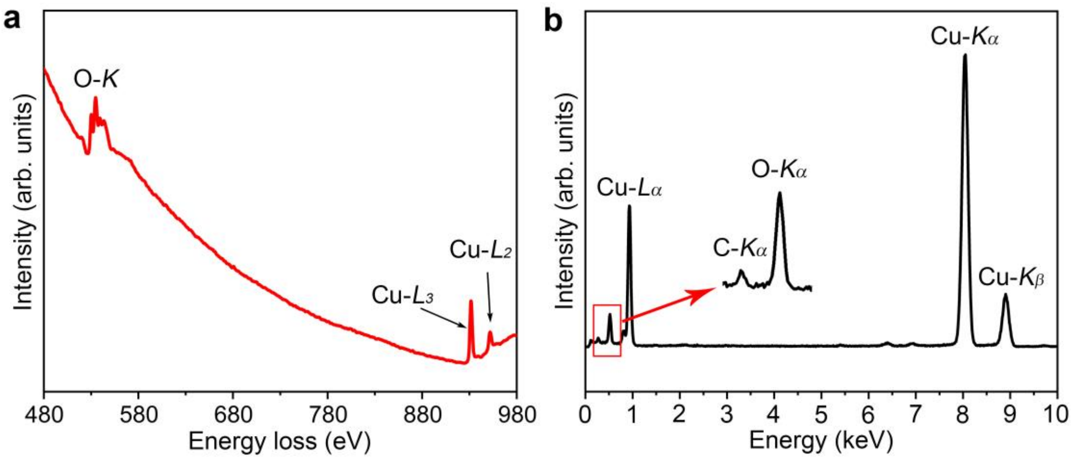

Supplementary Figure 5 | EELS and EDS spectra of the nucleated CuO_x phase.

a,**b**, EELS (**a**) and EDS (**b**) spectra of the nucleated CuO_x phase.

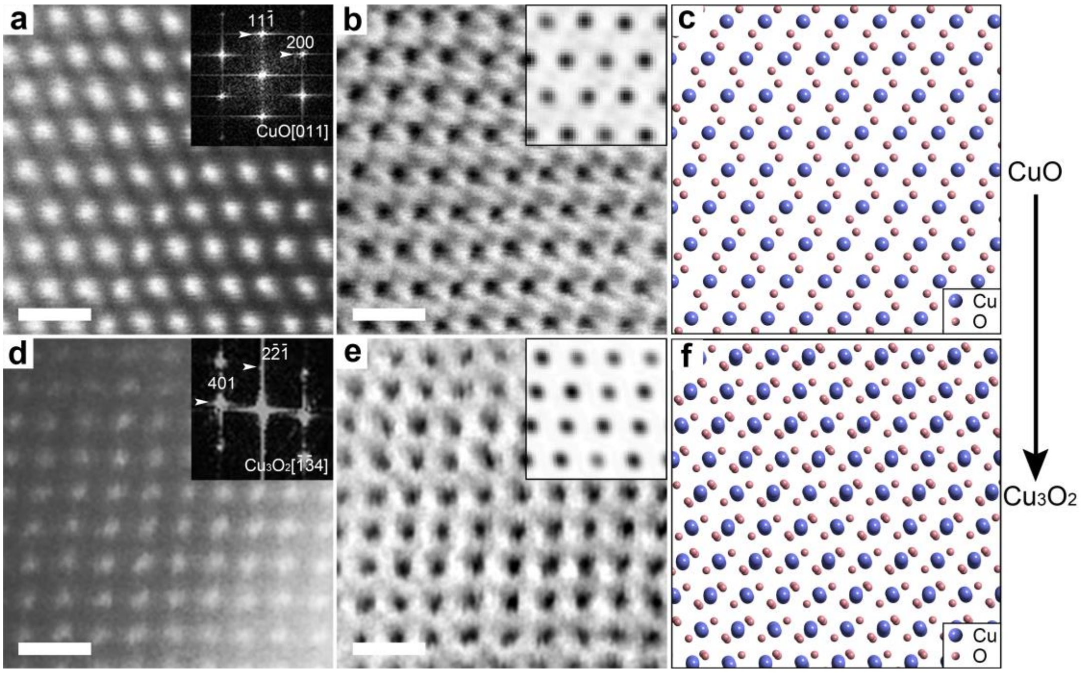

Supplementary Figure 6 | Structural characterization of CuO and Cu_3O_2 phases along $[011]_{CuO}$ // $[\bar{1}\bar{3}4]_{Cu_3O_2}$. a-c, HAADF, ABF images, and structural model of pristine CuO structure along $[011]_{CuO}$ axis, correspondingly. **d-f**, HAADF, ABF images, and structural model of Cu_3O_2 structure along $[\bar{1}\bar{3}4]_{Cu_3O_2}$ axis, respectively. The insets in **b** and **e** show the corresponding simulated ABF images. Scale bar, 0.5 nm.

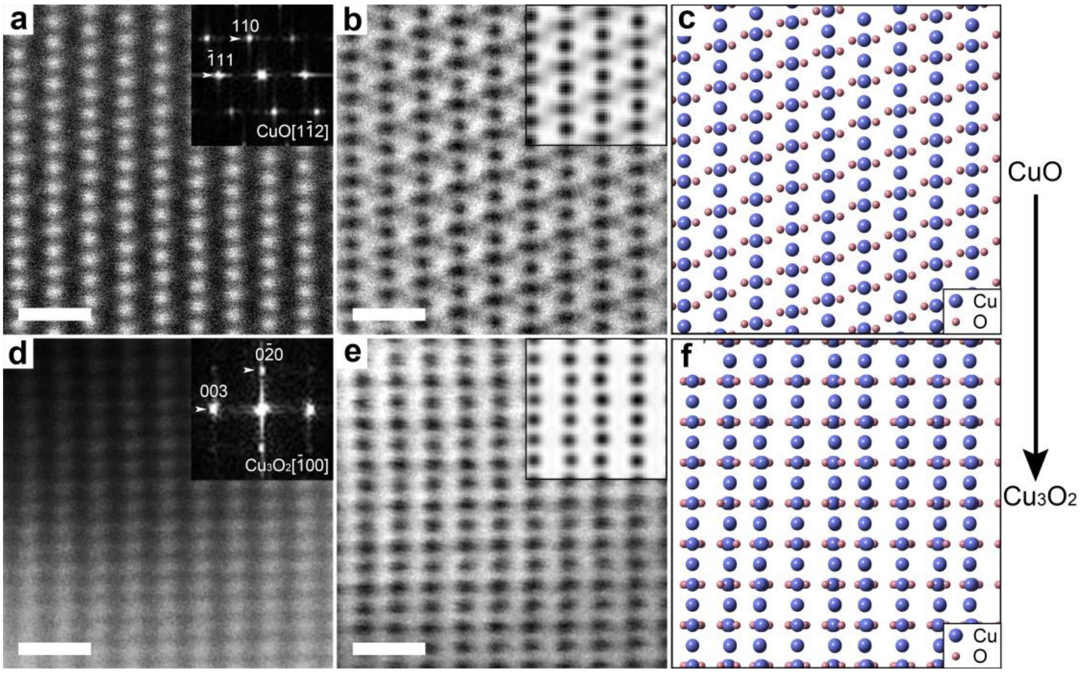

Supplementary Figure 7 | Structural characterization of CuO and Cu_3O_2 phases along $[1\bar{1}2]_{CuO}$ // $[\bar{1}00]_{Cu_3O_2}$. **a-c**, HAADF, ABF images, and structural model of pristine CuO structure along $[1\bar{1}2]_{CuO}$ axis, correspondingly. **d-f**, HAADF, ABF images, and structural model of Cu_3O_2 structure along $[\bar{1}00]_{Cu_3O_2}$ axis, respectively. The insets in **b** and **e** show the corresponding simulated ABF images. Scale bar, 0.5 nm.

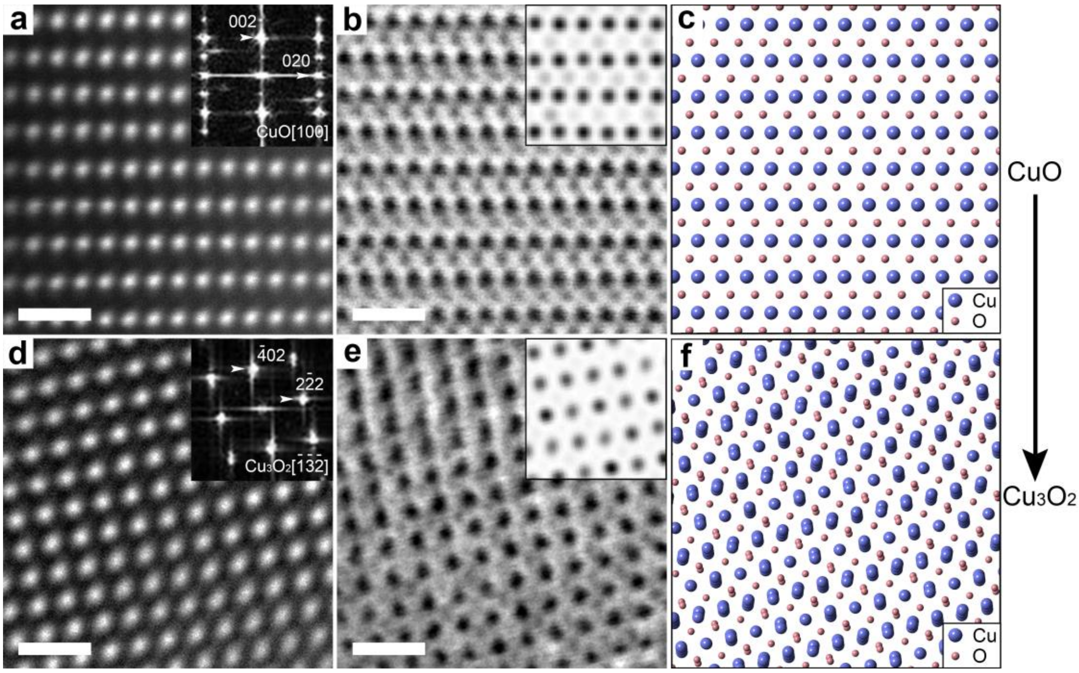

Supplementary Figure 8 | Structural characterization of CuO and Cu_3O_2 phases along $[100]_{CuO}//[\bar{1}\bar{3}\bar{2}]_{Cu_3O_2}$. **a-c**, HAADF, ABF images, and structural model of pristine CuO structure along $[100]_{CuO}$ axis, correspondingly. **d-f**, HAADF, ABF images, and structural model of Cu_3O_2 structure along $[\bar{1}\bar{3}\bar{2}]_{Cu_3O_2}$ axis, respectively. The insets in **b** and **e** show the corresponding simulated ABF images. Scale bar, 0.5 nm.

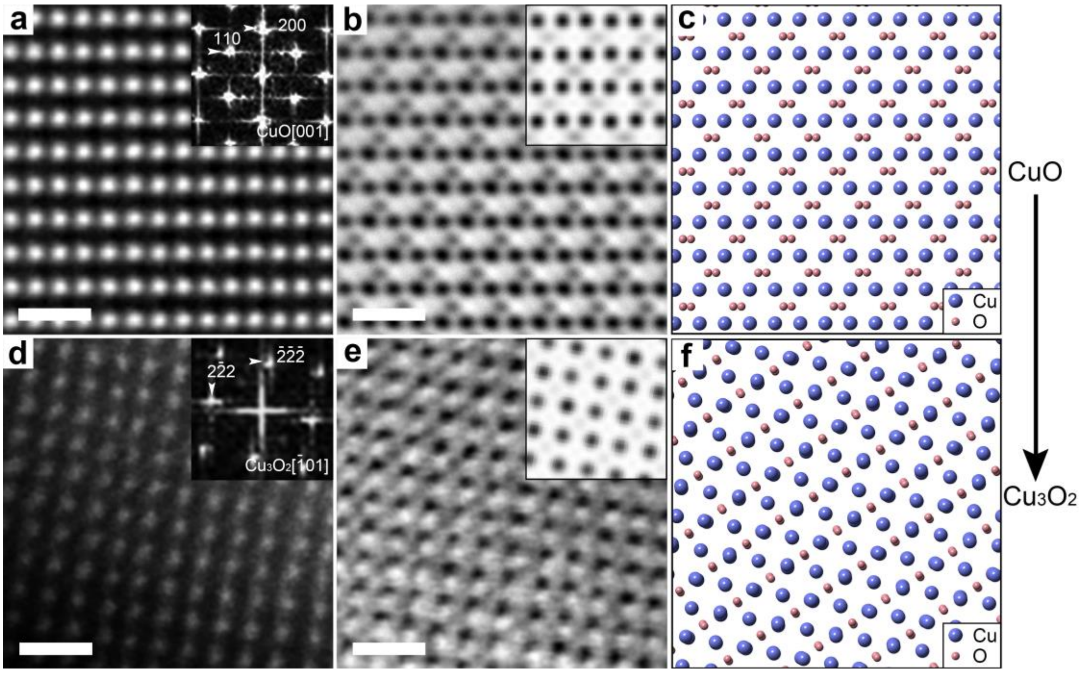

Supplementary Figure 9 | Structural characterization of CuO and Cu_3O_2 phases along $[001]_{CuO}$ // $[\bar{1}01]_{Cu_3O_2}$. **a-c**, HAADF, ABF images, and structural model of pristine CuO structure along $[001]_{CuO}$ axis, correspondingly. **d-f**, HAADF, ABF images, and structural model of Cu_3O_2 structure along $[\bar{1}01]_{Cu_3O_2}$ axis, respectively. The insets in **b** and **e** show the corresponding simulated ABF images. Scale bar, 0.5 nm.

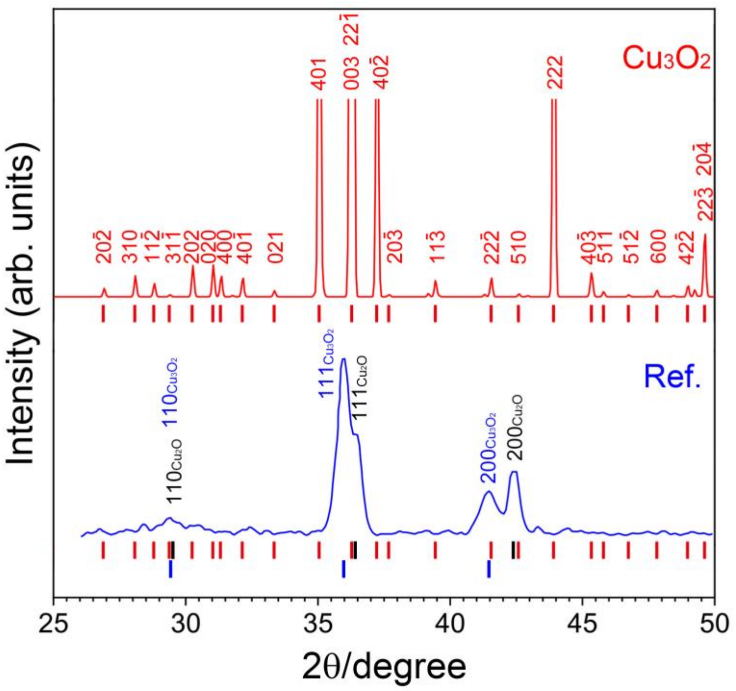

Supplementary Figure 10 | Experimental XRD pattern of the reported Cu_3O_2 and Cu_2O (blue line) (Cu_2O and Cu_3O_2 peaks are presented by black and blue vertical lines, respectively) in reference[1] and the simulated XRD pattern based on the monoclinic Cu_3O_2 structure (red vertical lines) in this paper.

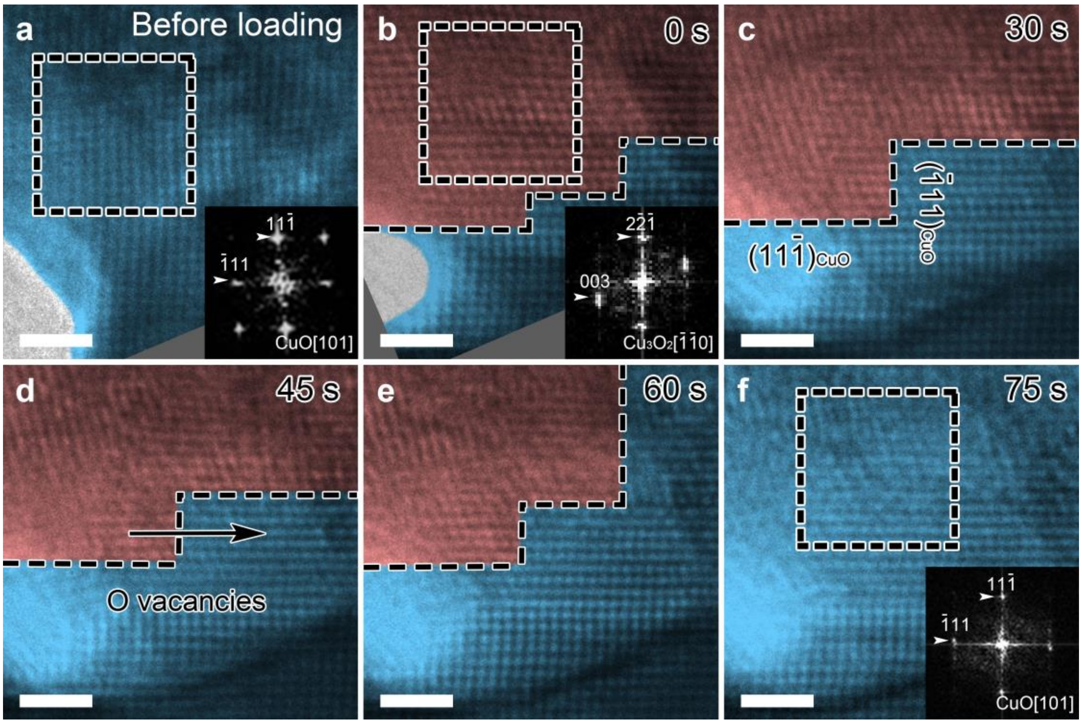

Supplementary Figure 11 | The anelastic behavior in CuO NW along $[101]_{CuO}$ axis. **a**, HRTEM image of the CuO matrix along $[101]_{CuO}$ axis before bending. **b**, The nucleation of CuO_x phase (inserted FFT pattern) at the moment after the stress is released. **c**-**f**, The phase transition from CuO_x phase to CuO (inserted FFT pattern in **f**), the black dotted curves point out the phase boundary. Scale bar, 2 nm.

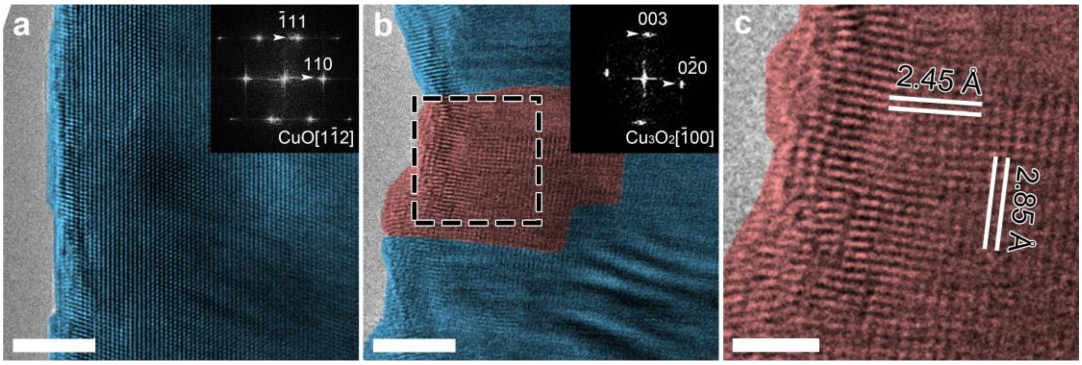

Supplementary Figure 12 | The anelastic behavior in CuO NW along $[1\bar{1}2]_{CuO}$ axis. **a**, HRTEM image of the CuO matrix along $[1\bar{1}2]_{CuO}$ axis before bending. **b**, The nucleation of CuO_x phase (inserted FFT pattern) the moment after the stress is released. **c**, Magnified image showing the CuO_x phase enclosed in **b**. Scale bar, 5 nm (**a**,**b**), 2 nm (**c**).

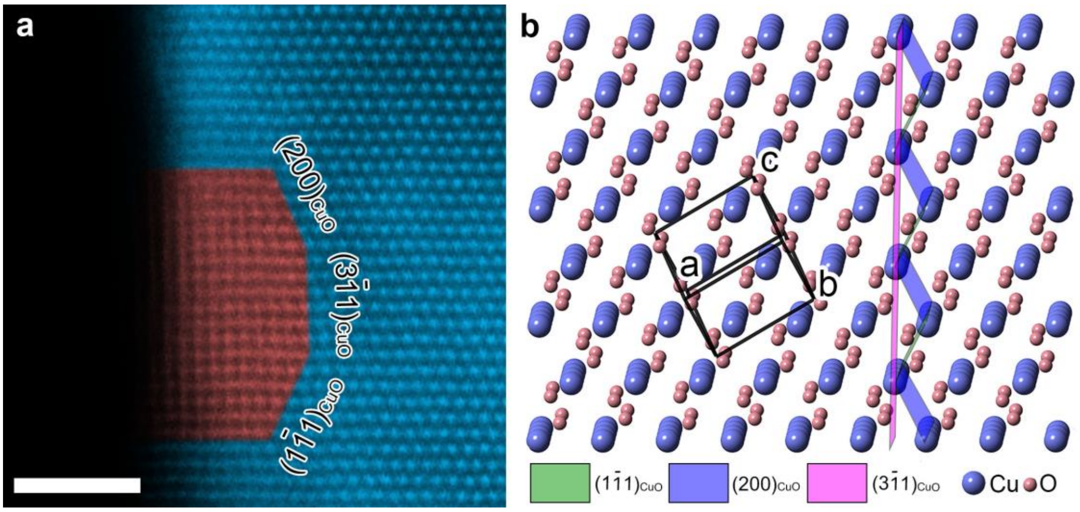

Supplementary Figure 13 | Illustration of $(3\bar{1}1)_{CuO}$, $(1\bar{1}1)_{CuO}$, and $(200)_{CuO}$ planes in CuO. a, HAADF image showing the boundary of CuO_x phase and CuO. **b**, Structural model of CuO showing that $(3\bar{1}1)_{CuO}$ is consisted of alternative $(200)_{CuO}$ and $(1\bar{1}1)_{CuO}$ planes. Scale bar, 2 nm (**a**).

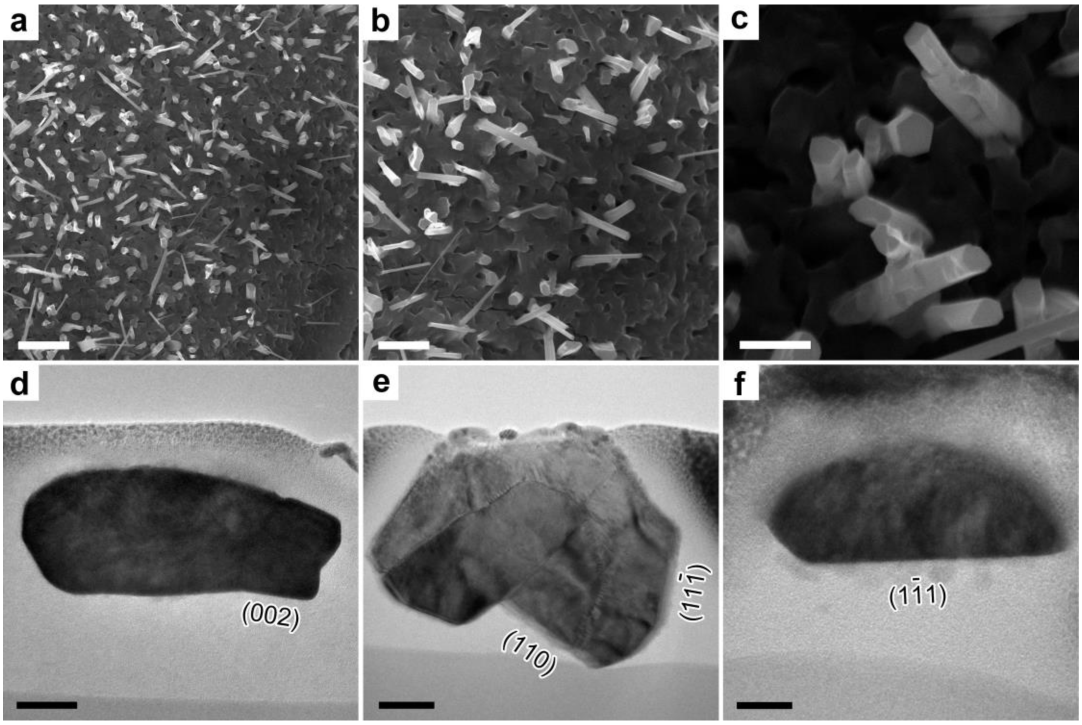

Supplementary Figure 14 | The morphologies of CuO NWs. a-c Scanning electron microscopy (SEM) images showing the morphologies of fabricated CuO NWs. **d-f**, TEM images showing the cross-sections of the CuO NWs fabricated by focused ion beam (FIB). Scale bar, 2 μm (**a**), 1 μm (**b**), 200 nm (**c**), 50 nm (**d**,**e**), 20 nm (**f**).

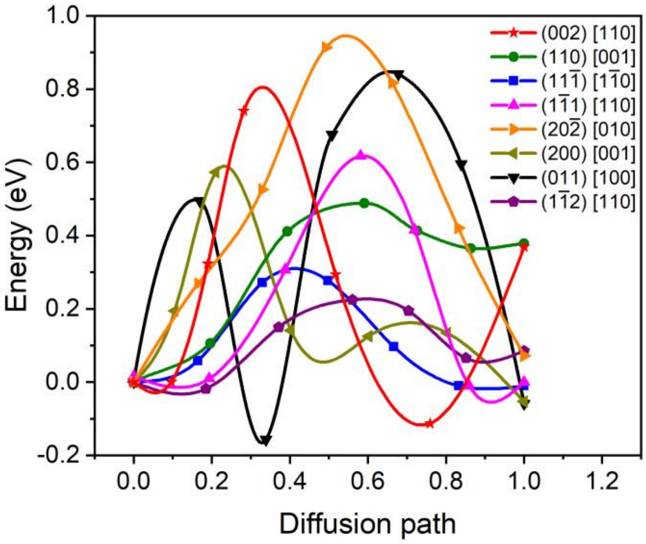

Supplementary Figure 15 | CI-NEB calculated diffusion energy barriers of oxygen vacancies on CuO surface planes including (002), (110), $(11\bar{1})$, $(1\bar{1}1)$, $(20\bar{2})$, (200), (011), and $(1\bar{1}2)$.

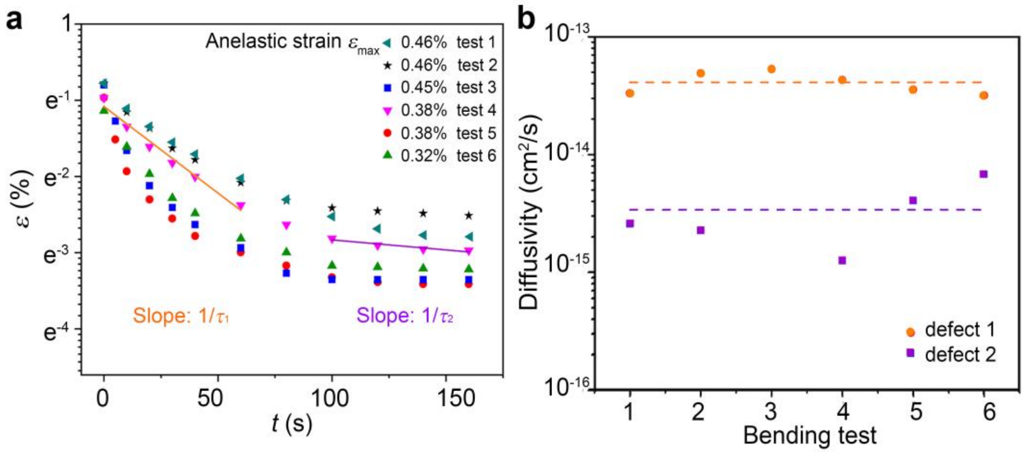

Supplementary Figure 16 | Estimation of diffusivity of defects in a single crystalline CuO NW (diameter ~46 nm). **a**, Anelastic strain (with logarithm transform for *y*-coordinate) as a function of time in six bending tests. Piecewise linear fitting conducted on all six bending tests. The slope equals to $1/\tau$. **b**, Diffusivities of two types of point defects. The orange and purple dashed lines indicate the averaged diffusivities of defects 1 and 2, respectively.

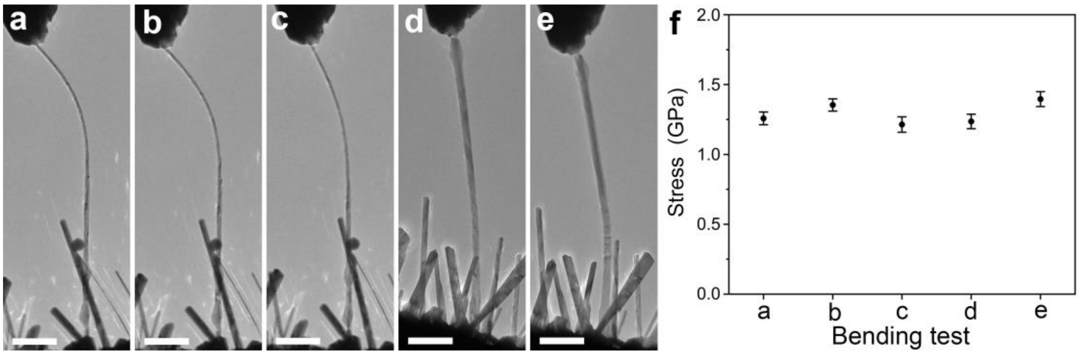

Supplementary Figure 17 | Bending stress of single crystalline CuO NWs. a-e, TEM images showing single crystalline CuO NW at the moment before W tip removed. **f**, Bending stress of five individual NWs before W tip removed. Error bars indicate the standard error of the mean (for 5 times of measurements). Scale bar, 500 nm (**a-e**).

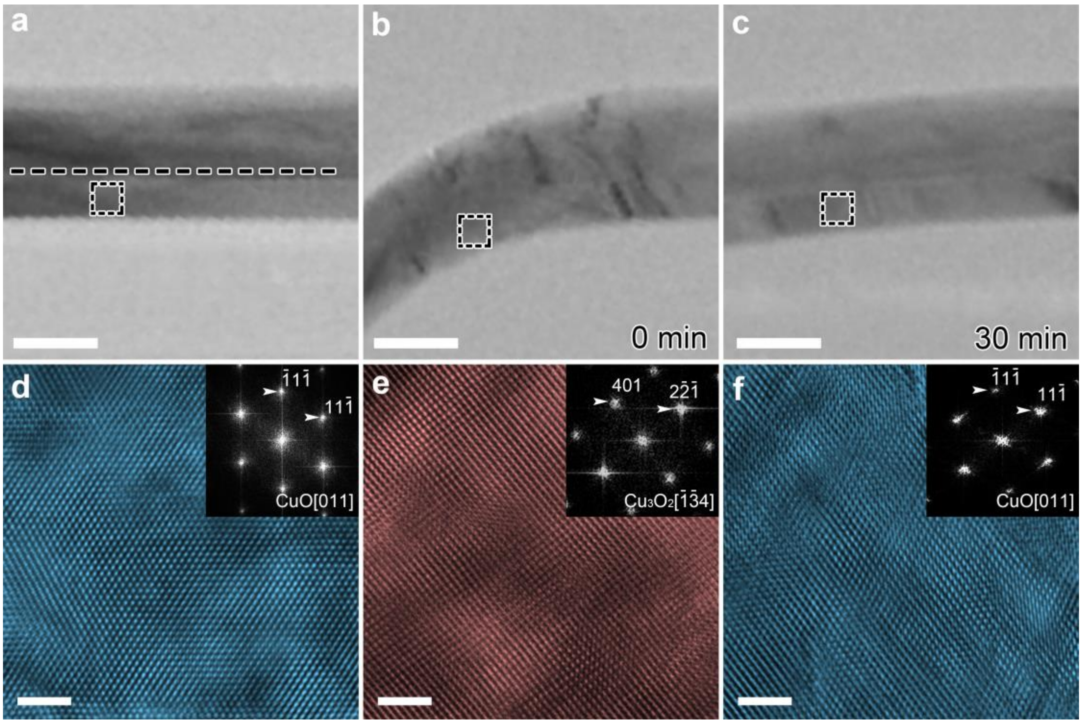

Supplementary Figure 18 | Oxygen-vacancy migration induced phase transition in a twinned CuO NW. a,**b**, Low-magnified TEM images of the twinned CuO NW before (**a**) and after (**b**) bending test, dotted line in **a** shows the twin boundary. **c**, TEM image of the NW relaxed for 30 minutes. **d**-**f**, HRTEM images of the enclosed area in (**a**-**c**) respectively. Scale bar, 50 nm (**a**-**c**), 2 nm (**d**-**f**).

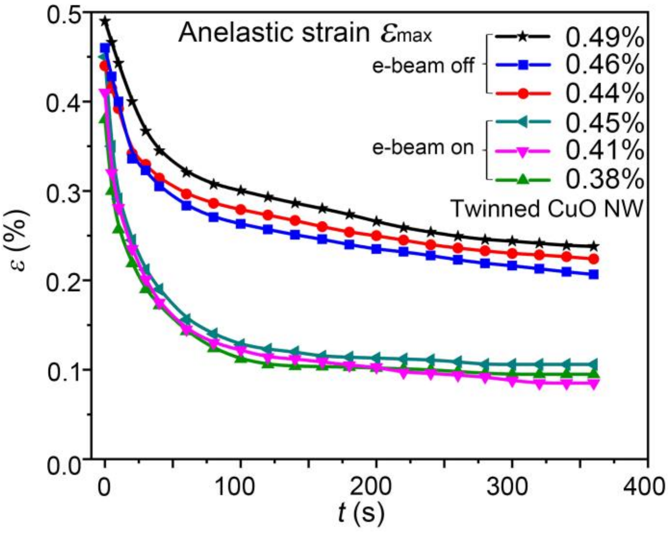

Supplementary Figure 19 | Anelasticity in a twinned CuO NW. Anelastic strain recovery as a function of time in three bending tests with and without e-beam irradiation on the same twinned CuO NW, respectively. Without e-beam irradiation, the time-interval for the image recording is 20 seconds.

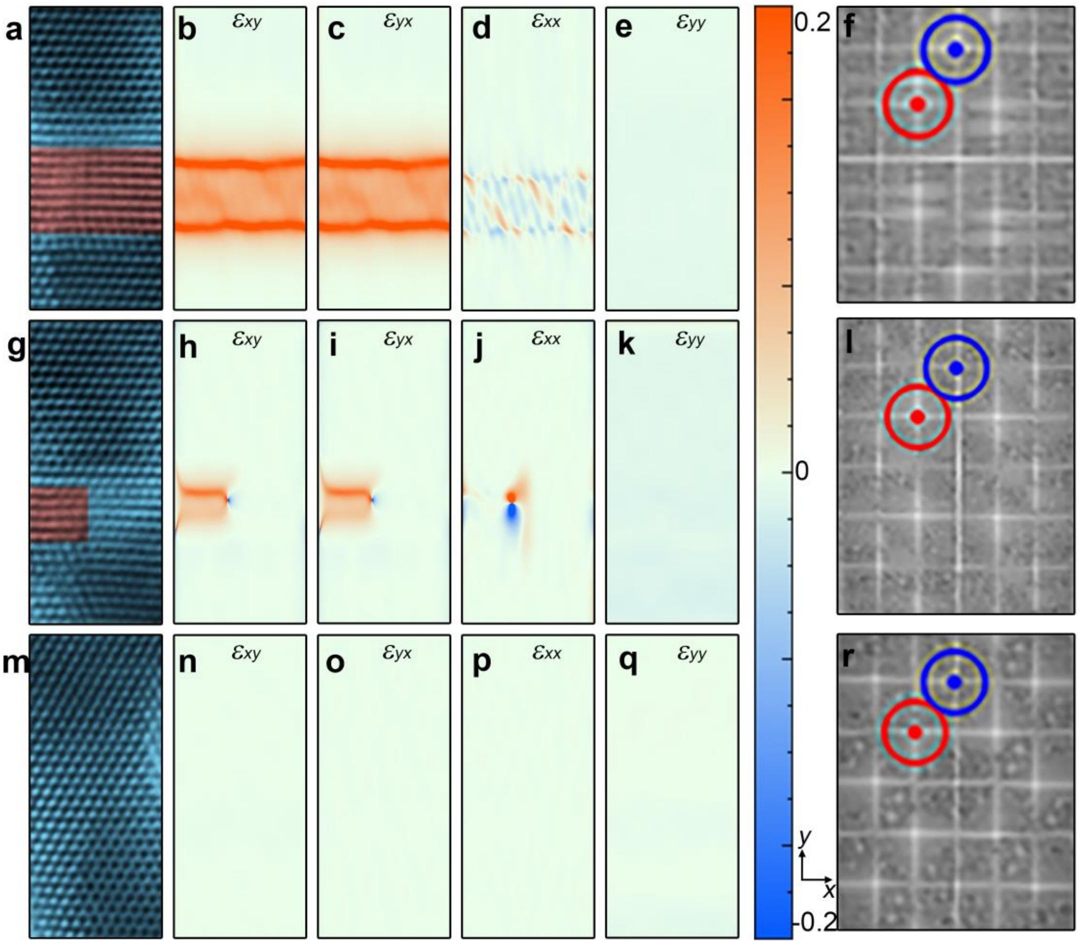

Supplementary Figure 20 | The strain analysis based on GPA along [011]$_{CuO}$ axis. a,**g**,**m**, Sequential HRTEM images showing the transition of CuO_x phase back to CuO as shown in Figs. 4d-f. **b-e**, **h-k** and **n-q**, The four components ε_{xy}, ε_{yx}, ε_{xx} and ε_{yy} of the corresponding strain map in **a**, **g** and **m**. **f**,**l**,**r**, Two nonlinear g vectors in the reciprocal space are used for the GPA analysis in **a**, **g** and **m**.

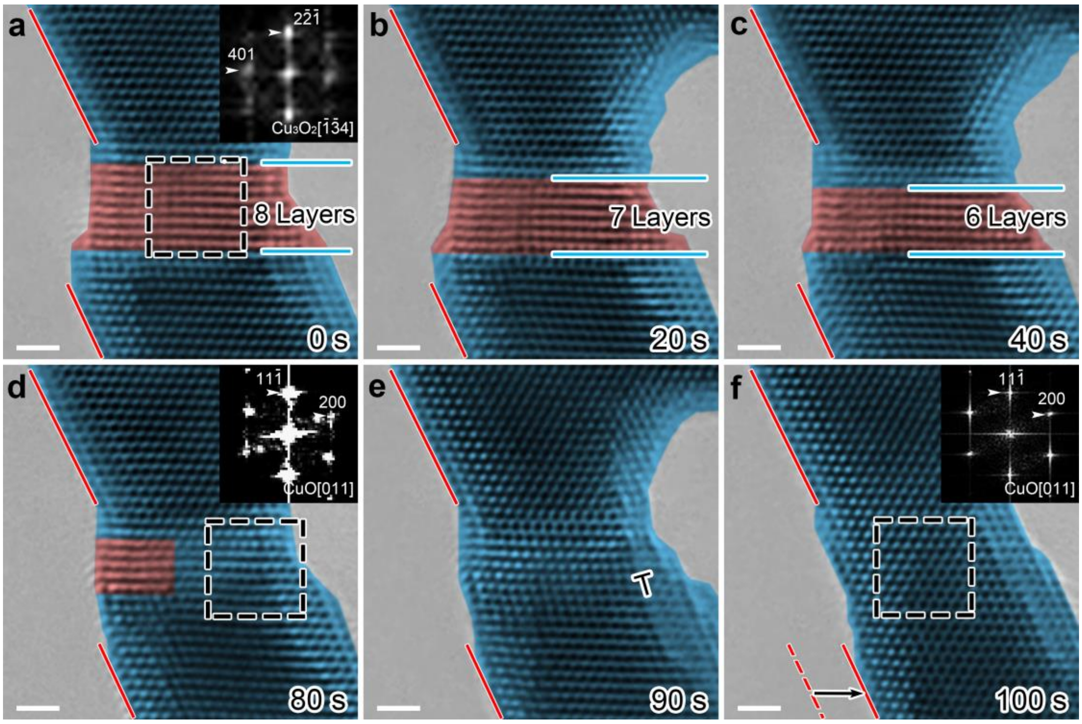

Supplementary Figure 21 | The strain evolution induced by the phase transition.

a-f, HRTEM images showing the transformation from CuO_x phase to CuO along the $[011]_{CuO}$ axis. The change of shear strain is indicated by the red lines. Scale bar, 1 nm.

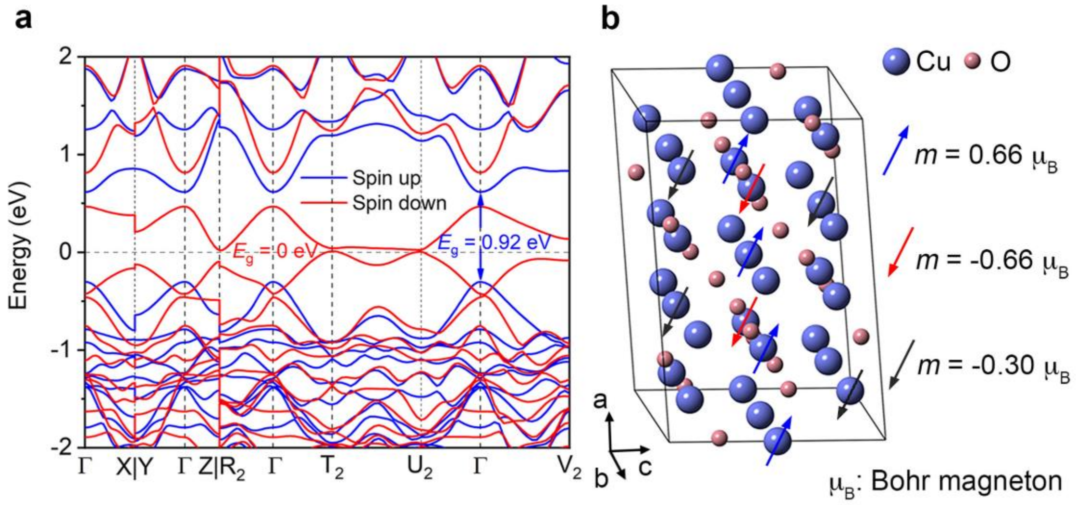

Supplementary Figure 22 | Calculated electronic band structure and antiferromagnetic (AFM) ordering of Cu_3O_2. a, Spin-polarized PBE+*U* calculated band of Cu_3O_2 shows that the spin up channel has a 0.92 eV band gap and the spin down channel is gapless, which reveals the half-metallic nature of Cu_3O_2. **b**, AFM ordering of Cu in Cu_3O_2 confirmed by first-principles calculations.

Supplementary Table 1. Atom positions in monoclinic Cu_3O_2 phase

Cu_3O_2			a = 11.49 Å	b = 5.75 Å	c = 7.49 Å
Space group: $C2/m$			$\alpha = 90^o$	$\beta = 97.18^o$	$\gamma = 90^o$
Atoms	Occupancy	Wyckoff position	Coordinates		
Cu	1	8j	0.093	0.242	0.142
Cu	1	4i	0.846	0.000	0.147
Cu	1	4i	0.835	0.500	0.168
Cu	1	4f	0.750	0.750	0.500
Cu	1	2d	0.000	0.500	0.500
Cu	1	2c	0.000	0.000	0.500
O	1	4i	0.843	0.500	0.415
O	1	4i	0.839	0.000	0.432
O	1	4i	0.822	0.500	0.920
O	1	4i	0.011	0.000	0.232

Supplementary References

1. Farhad, S. F. U., Webster, R. F. & Cherns, D. Electron microscopy and diffraction studies of pulsed laser deposited cuprous oxide thin films grown at low substrate temperatures. *Materialia* **3**, 230-238 (2018).
