# Supplementary material for: Room-temperature oxygen vacancy migration induced reversible phase transformation during the anelastic deformation in CuO
Source: Nat Commun. 2021 Jun 23;12:3863. doi: 10.1038/s41467-021-24155-z (PMC8222270; doi:10.1038/s41467-021-24155-z)
Supplement: Supplementary file 3 — Description of Additional Supplementary Files [file 41467_2021_24155_MOESM3_ESM.pdf]

Description of Additional Supplementary Files

File Name: Supplementary Movie 1
Description: The bending deformation and anelastic strain recovery of a single crystalline CuO NW, played at 20× speed.

File Name: Supplementary Movie 2
Description: The atomistic view of the lattice evolution during the CuO_x-CuO phase transition along $[011]_{CuO} // [\bar{1}\bar{3}4]_{Cu_3O_2}$, played at 20× speed.

File Name: Supplementary Movie 3
Description: The dynamic HAADF images showing the phase boundary evolution during the CuO_x-CuO phase transition along $[011]_{CuO} // [\bar{1}\bar{3}4]_{Cu_3O_2}$, played at 15× speed.

File Name: Supplementary Movie 4
Description: The strain evolution associated with the CuO_x-CuO phase transition along $[011]_{CuO} // [\bar{1}\bar{3}4]_{Cu_3O_2}$, played at 10× speed.
